# Supplementary material for: Single-cell analysis of chromatin accessibility in the adult mouse brain
Source: Nature. 2023 Dec 13;624(7991):378–89. doi: 10.1038/s41586-023-06824-9 (PMC10719105; doi:10.1038/s41586-023-06824-9)
Supplement: Supplementary file 1 — Reporting Summary [file 41586_2023_6824_MOESM1_ESM.pdf]

nature portfolio

Corresponding author(s): Bing Ren

Last updated by author(s): Oct 30, 2023

Reporting Summary

Nature Portfolio wishes to improve the reproducibility of the work that we publish. This form provides structure for consistency and transparency in reporting. For further information on Nature Portfolio policies, see our Editorial Policies and the Editorial Policy Checklist.

Statistics

For all statistical analyses, confirm that the following items are present in the figure legend, table legend, main text, or Methods section.

n/a	Confirmed	
☐	☒	The exact sample size (*n*) for each experimental group/condition, given as a discrete number and unit of measurement
☐	☒	A statement on whether measurements were taken from distinct samples or whether the same sample was measured repeatedly
☐	☒	The statistical test(s) used AND whether they are one- or two-sided<br>*Only common tests should be described solely by name; describe more complex techniques in the Methods section.*
☐	☒	A description of all covariates tested
☐	☒	A description of any assumptions or corrections, such as tests of normality and adjustment for multiple comparisons
☐	☒	A full description of the statistical parameters including central tendency (e.g. means) or other basic estimates (e.g. regression coefficient) AND variation (e.g. standard deviation) or associated estimates of uncertainty (e.g. confidence intervals)
☐	☒	For null hypothesis testing, the test statistic (e.g. *F*, *t*, *r*) with confidence intervals, effect sizes, degrees of freedom and *P* value noted<br>*Give P values as exact values whenever suitable.*
☒	☐	For Bayesian analysis, information on the choice of priors and Markov chain Monte Carlo settings
☐	☒	For hierarchical and complex designs, identification of the appropriate level for tests and full reporting of outcomes
☐	☒	Estimates of effect sizes (e.g. Cohen's *d*, Pearson's *r*), indicating how they were calculated

Our web collection on statistics for biologists contains articles on many of the points above.

Software and code

Policy information about availability of computer code

Data collection	Sony Cell Sorter Software v2.1.2-5, Biomek Software 5.1 (library preparation), Illumina HiSeq2500, HiSeq4000, and NovaSeq 6000 instrument control software (sequencing)
Data analysis	bwa (v.0.7.17), HOMER(v4.11), BEDTools (v2.25.0), MACS2 (v2.1.2), GNU parallel (20220822),<br>GNU R (v4.3.1), ggplot2(3.4.3), stringr (1.5.0), purrr(1.0.2), dplyr (1.1.3), Seurat v5,<br>Python (v3.10), SnapATAC2 (v2.4), Sklearn(v1.1.0), Cicero (v3.16), CellOracle (v0.15.0),<br>Sony SH800S software,<br>https://github.com/beyondpie/CEMBA_wmb_snATAC

For manuscripts utilizing custom algorithms or software that are central to the research but not yet described in published literature, software must be made available to editors and reviewers. We strongly encourage code deposition in a community repository (e.g. GitHub). See the Nature Portfolio guidelines for submitting code & software for further information.

Data

Policy information about availability of data

All manuscripts must include a data availability statement. This statement should provide the following information, where applicable:

- Accession codes, unique identifiers, or web links for publicly available datasets
- A description of any restrictions on data availability
- For clinical datasets or third party data, please ensure that the statement adheres to our policy

Demultiplexed FASTQ files are available at the NEMO archive (NEMO, RRID: SCR_016152) at https://assets.nemoarchive.org/dat-bej4ymm (the raw directory under the source data URL in this archive), and at the NCBI under GEO accession number GSE246791 . Processed data are available at our web portal (http://www.catlas.org) and the same GEO accession number above.

Research involving human participants, their data, or biological material

Policy information about studies with human participants or human data. See also policy information about sex, gender (identity/presentation), and sexual orientation and race, ethnicity and racism.

Reporting on sex and gender	N/A
Reporting on race, ethnicity, or other socially relevant groupings	N/A
Population characteristics	N/A
Recruitment	N/A
Ethics oversight	N/A

Note that full information on the approval of the study protocol must also be provided in the manuscript.

Field-specific reporting

Please select the one below that is the best fit for your research. If you are not sure, read the appropriate sections before making your selection.

☒ Life sciences ☐ Behavioural & social sciences ☐ Ecological, evolutionary & environmental sciences

For a reference copy of the document with all sections, see nature.com/documents/nr-reporting-summary-flat.pdf

Life sciences study design

All studies must disclose on these points even when the disclosure is negative.

Sample size	No statistical methods were used to predetermine sample size. For each of 117 regions from the mouse brain, dissected brain tissues were pooled from 2-31 (only 2 dissections from the mouse cerebellum region had 2 animals for snATAC-seq library construction, all the other samples had 4-31 animals) of the same sex to obtain enough nuclei for single nucleus ATAC-seq for each biological replica, and two biological replicas were performed. In total, 234 samples were included.
Data exclusions	No samples were excluded.<br>For analysis, only nuclei with >1,000 fragments / nucleus and transcription start site enrichment > 10 were selected.
Replication	Experiments were performed for 2 biological replicates for each of 117 dissection regions. All the replicates were successfully collected.
Randomization	There was no randomization of the samples. For each dissection region, dissected brain tissues were pooled from 2-31 (only 2 dissections from the mouse cerebellum region had 2 animals for snATAC-seq library construction, all the other samples had 4-31 animals) of the same sex. The 117 dissection regions were designed before experiments for analyzing the whole mouse brain in a comprehensive way.
Blinding	Investigators were not blinded to the specimen being investigated based on our experimental design above.

Reporting for specific materials, systems and methods

We require information from authors about some types of materials, experimental systems and methods used in many studies. Here, indicate whether each material, system or method listed is relevant to your study. If you are not sure if a list item applies to your research, read the appropriate section before selecting a response.

Materials & experimental systems

n/a	Involved in the study
☒	☐ Antibodies
☒	☐ Eukaryotic cell lines
☒	☐ Palaeontology and archaeology
☐	☒ Animals and other organisms
☒	☐ Clinical data
☒	☐ Dual use research of concern
☒	☐ Plants

Methods

n/a	Involved in the study
☒	☐ ChIP-seq
☐	☒ Flow cytometry
☒	☐ MRI-based neuroimaging

Animals and other research organisms

Policy information about studies involving animals; ARRIVE guidelines recommended for reporting animal research, and Sex and Gender in Research

Laboratory animals	Adult (P56) C57BL/6J male mice were purchased from Jackson Laboratories at seven weeks of age and maintained in the Salk animal barrier facility under a 12-h light/12-h dark cycle in a temperature-controlled room with ad libitum access to water and food until euthanasia. The temperature in the animal facility was maintained within the range of20 to 22.2C, while the humidity levels varied between 35 and 60%.
Wild animals	No wild animals were used in this study.
Reporting on sex	Only male mice were used.
Field-collected samples	No filed-collected samples were used in this study.
Ethics oversight	All experimental procedures using live animals were approved by the SALK Institute Animal Care and Use Committee under protocol number 18-00006.

Note that full information on the approval of the study protocol must also be provided in the manuscript.

Plants

Seed stocks	N/A
Novel plant genotypes	N/A
Authentication	N/A

Flow Cytometry

Plots

Confirm that:

- ☒ The axis labels state the marker and fluorochrome used (e.g. CD4-FITC).
- ☒ The axis scales are clearly visible. Include numbers along axes only for bottom left plot of group (a 'group' is an analysis of identical markers).
- ☒ All plots are contour plots with outliers or pseudocolor plots.
- ☒ A numerical value for number of cells or percentage (with statistics) is provided.

Methodology

Sample preparation	Nuclei were stained with DRAQ7 (#7406, Cell Signaling)
Instrument	Sony SH800
Software	Sony SH800S software

Cell population abundance	Cell populations within each sample were determined using snATAC-seq as described in the manuscript. See Methods and Supplementary table 2 and 3 for details.
Gating strategy	Potential nuclei were first identified using FSC-Area and BSC-Area. Next doublets were removed based on BSC and FSC signal width. DRAQQ7 postive nuclei with 2n count were sorted.

☒ Tick this box to confirm that a figure exemplifying the gating strategy is provided in the Supplementary Information.
