# Supplementary material for: Single-cell analysis of chromatin accessibility in the adult mouse brain
Source: Nature. 2023 Dec 13;624(7991):378–89. doi: 10.1038/s41586-023-06824-9 (PMC10719105; doi:10.1038/s41586-023-06824-9)
Supplement: Supplementary file 3 — Supplementary Tables 1–25 and their legends. [file 41586_2023_6824_MOESM3_ESM.zip › 2023-03-05453B-s3/SI Tables/2023-03-05453B-SI Guide.docx]

**Single-cell analysis of chromatin accessibility in adult mouse brain**

Songpeng Zu^1*^, Yang Eric Li^1*,9^, Kangli Wang^1*^, Ethan Armand^1^, Sainath Mamde^1^, Maria Luisa Amaral^1^, Yuelai Wang^1^, Andre Chu^1^, Yang Xie^1^, Michael Miller^2^, Jie Xu^1^, Zhaoning Wang^1^, Kai Zhang^1^, Bojing Jia^1^, Xiaomeng Hou^2^, Lin Lin^2^, Qian Yang^2^, Seoyeon Lee^1^, Bin Li^1^, Samantha Kuan^1^, Hanqing Liu^8^, Jingtian Zhou^8^, Antonio Pinto-Duarte^4^, Jacinta Lucero^4^, Julia Osteen^4^, Michael Nunn^3^, Kimberly A. Smith^5^, Bosiljka Tasic^5^, Zizhen Yao^5^, Hongkui Zeng^5^, Zihan Wang^6^, Jingbo Shang^6^, M. Margarita Behrens^4^, Joseph R. Ecker^3^, Allen Wang^2^, Sebastian Preissl^2,7^, Bing Ren^1,2,@^

^1^ Department of Cellular and Molecular Medicine, University of California San Diego, School of Medicine, La Jolla, CA, USA

^2^ Center for Epigenomics, University of California San Diego, School of Medicine, La Jolla, CA, USA

^3^ Howard Hughes Medical Institute, The Salk Institute for Biological Studies, La Jolla, CA, USA

^4^ The Salk Institute for Biological Studies, La Jolla, CA, USA

^5^ Allen Institute for Brain Science, Seattle, WA 98109, USA

^6^ Department of Computer Science and Engineering, University of California San Diego, La Jolla, CA, USA

^7^ Institute of Experimental and Clinical Pharmacology and Toxicology, Faculty of Medicine, University of Freiburg, Freiburg, Germany

^8^ Genomic Analysis Laboratory, The Salk Institute for Biological Studies, CA, USA

^9^ Department of Neurosurgery and Genetics, Washington University School of Medicine, St. Louis, MO 63110, USA

* These authors contributed equally

^@^ Correspondence: biren@health.ucsd.edu

**Supplementary Tables**

**Supplementary Table 1**: Sample and dissection summary

**Supplementary Table 2**: Metadata table for all the 2.3 million nuclei in the snATAC-seq data

**Supplementary Table 3**: Metadata table for clustering, including resolution parameters and full name of the annotations in Figure 1c UMAP

**Supplementary Table 4**: L4-level annotation based on the integration analysis with scRNA-seq data including major region information

**Supplementary Table 5**: Transfer label scores for the integration of the snATAC-seq with the scRNA-seq data. Both cluster and subclass level scores are presented

**Supplementary Table 6**: Cell-subtype specific cCREs

**Supplementary Table 7**: List of the genomic locations of all the cCREs

**Supplementary Table 8**: List of number of cCREs per cell subtype and cell subclass

**Supplementary Table 9**: List of cCREs having no overlaps with the ENCODE DHSs

**Supplementary Table 10**: Module assignments of all the cCREs in the non-negative matrix factorization

**Supplementary Table 11**: Association of *cis* regulatory modules with cell subclasses

**Supplementary Table 12**: Summary of all the co-accessible cCRE pairs

**Supplementary Table 13**: Summary of all the gene-cCRE correlations

**Supplementary Table 14**: Module assignment of the putative enhancers in the non-negative matrix factorization

**Supplementary Table 15**: Module assignment of the cell subclasses used in the putative enhancer module analysis

**Supplementary Table 16**: Known motif enrichment in putative enhancers

**Supplementary Table 17**: Summary of gene regulatory networks in all the cell subclasses

**Supplementary Table 18**: Counts of the network motifs in all the cell-subclass gene regulatory networks

**Supplementary Table 19**: Counts of the network motifs in the mouse brain regions

**Supplementary Table 20**: Region information of the cell subclasses for network motif analysis

**Supplementary Table 21:** The eigen-vector centrality scores for 403 transcription factors in each of the 267 cell subclasses based on their gene regulatory networks

**Supplementary Table 22**: GO analysis of the genes positively correlated with TE-cCREs

**Supplementary Table 23**: Differential chromatin accessibility of transposable-element related cCREs correlated with synaptic genes

**Supplementary Table 24**: Motif enrichment of differentially accessible transposable-element related cCREs.

**Supplementary Table 25**: Deep learning model performance on cell types.
